# Supplementary material for: Association of Homocysteine, S-Adenosylhomocysteine and S-Adenosylmethionine with Cardiovascular Events in Chronic Kidney Disease
Source: Nutrients. 2025 Feb 10;17(4):626. doi: 10.3390/nu17040626 (PMC11858042; doi:10.3390/nu17040626)
Supplement: Supplementary file 1 [file nutrients-17-00626-s001.zip › nutrients-3391304-supplementary.pdf]

Inclusion criteria

- CKD KDIGO category G 2 to G4 (eGFR 89 – 15 ml/min/1.73 m² according to the 2009 creatinine-based CKD-EPI equation)
- in case of CKD KDIGO category G2 (eGFR 89 – 60 ml/min/1.73 m²) one of the following markers of kidney damage was needed to enter the study: proteinuria ≥ 300 mg/g, albuminuria ≥ 17 mg/g in men and ≥ 25 mg/g in women, persistent glomerular hematuria, elevated plasma cystatin C levels (> 1.05 mg/l), elevated plasma creatinine levels (> 1.2 mg/dl in men and > 0.9 mg/dl in women), a tubulopathy and/or histopathological findings that proven kidney disease

Exclusion criteria

- CKD KDIGO category G1 or G5 (eGFR > 90 ml/min/1.73 m² or < 15 ml/min/1.73 m² according to the 2009 creatinine-based CKD-EPI equation)
- systemic immunosuppressive treatment
- a known infection with human immunodeficiency virus
- an elevated, asymptomatic elevation of C-reactive protein > 50 mg/l
- a clinical apparent infection with fever and/or necessity to treat with antibiotics
- known, active cancer
- acute kidney injury (creatinine elevation of more than 50 % in the last four weeks prior to study inclusion)

Table S1: Baseline characteristics stratified according to MACE

	MACE		
	**YES (n = 55)**	**NO (n = 256)**	**p**
Patient demographics			
Age [years]	76.2 [68.3; 81.7]	67.8 [58.8; 75.7]	**<0.001**
Gender [female]	19 [34.5 %]	106 [41.4 %]	0.348
Smoking [yes]	7 [12.7 %]	26 [10.2 %]	0.576
CVD [prevalent]	27 [49.1 %]	72 [28.1 %]	**0.002**
Diabetes mellitus [yes]	26 [47.3 %]	85 [33.2 %]	**0.048**
BMI [kg/m^2]	29 [26; 35]	30 [27; 34]	0.454
SBP [mmHg]	152 [131; 165]	142 [132; 158]	0.200
Total cholesterol [mg/dl]	170 [142; 207]	177 [155; 205]	0.522
Renal parameters			
eGFR [ml/min/1.73 m^2]	34.8 [22.7; 48.5]	46.3 [33.8; 59.4]	**<0.001**
Albuminuria [mg/g crea]	110 [20; 440]	30 [10; 140]	**0.003**
One-carbon metabolites			
SAH [nmol/l]	58.1 [38.0; 86.0]	37.5 [26.2; 55.3]	**0.002**
SAM [nmol/l]	198.0 [172.5; 249.4]	180.5 [149.1; 216.6]	**0.007**
Homocysteine [µmol/l]	19.8 [14.9; 23.9]	16.3 [13.4; 20.6]	**0.020**

Table S2: Interrelationship of the concentrations of C1 metabolites and smoking, gender and diabetes mellitus.

		SAH	SAM	Homocysteine	SAM / SAH
Active smoker	yes	46.6 ± 34.0	193.2 ± 69.6	17.2 ± 5.8	5.7 ± 2.9
	no	50.6 ± 36.4	193.4 ± 55.3	18.6 ± 7.3	5.0 ± 2.7
	p-value	0.554	0.984	0.316	0.162
Gender	yes	46.3 ± 31.0	192.4 ± 54.6	17.5 ± 7.1	5.3 ± 2.5
	no	52.6 ± 39.0	194.0 ± 58.5	19.0 ± 7.1	5.0 ± 2.9
	p-value	0.140	0.812	0.081	0.222
Diabetes mellitus	yes	52.4 ± 37.2	205.5 ± 56.7	18.7 ± 6.4	5.2 ± 2.8
	no	48.9 ± 35.5	186.7 ± 56.0	18.3 ± 7.5	5.0 ± 2.7
	p-value	0.429	**0.006****	0.651	0.596

*p < 0.05; **p < 0.01; ***p < 0.001. Correlation coefficients with p-values below 0.05 are given in bold letters.

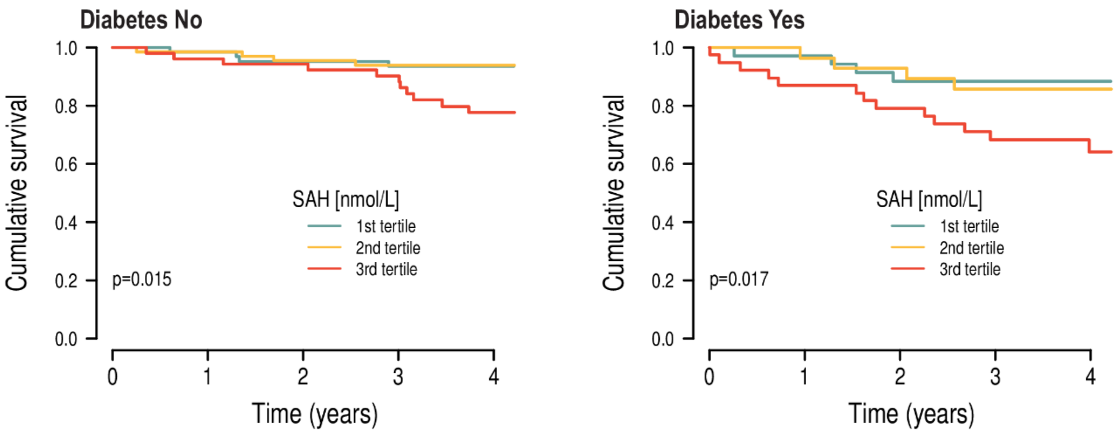

Figure S1: After stratifying the study cohort by their plasma SAH levels into tertiles, higher plasma SAH levels were significantly associated with the primary endpoint in univariate Kaplan-Meier survival analysis in non-diabetes (p = 0.015) and in diabetes (p = 0.017).

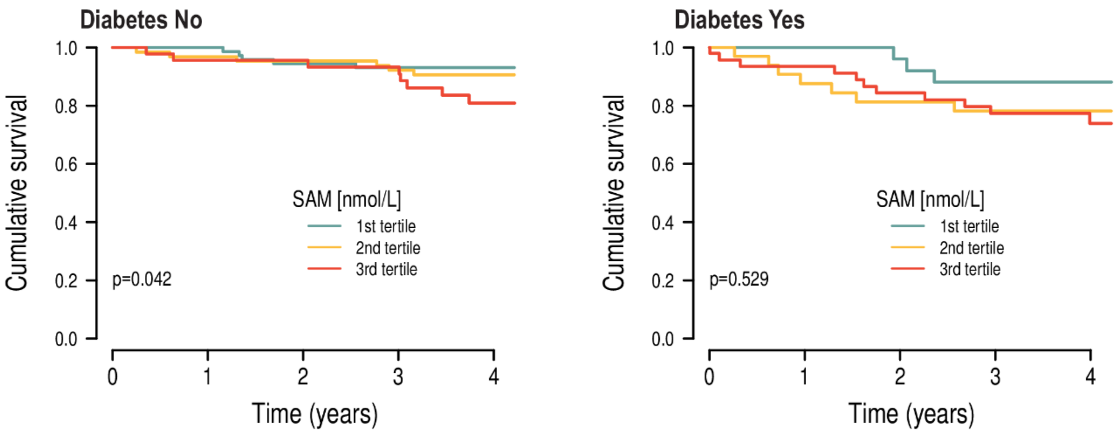

Figure S2: After stratifying the study cohort by their plasma SAM levels into tertiles, higher plasma SAM levels were significantly associated with the primary endpoint in univariate Kaplan-Meier survival analysis in non-diabetes (p = 0.042), but not in diabetes (p = 0.529).

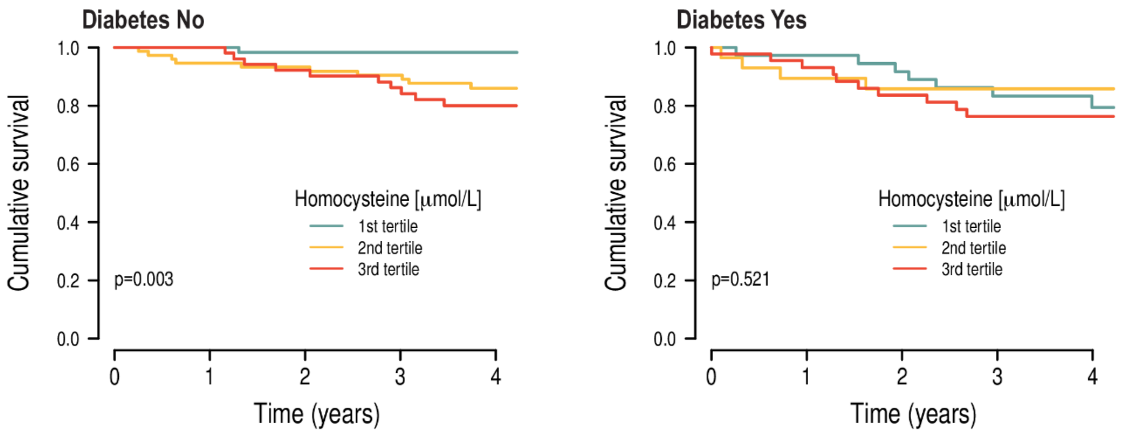

Figure S3: After stratifying the study cohort by their plasma homocysteine levels into tertiles, higher plasma homocysteine levels were significantly associated with the primary endpoint in univariate Kaplan-Meier survival analysis in non-diabetes (p = 0.003), but not in diabetes (p = 0.521).

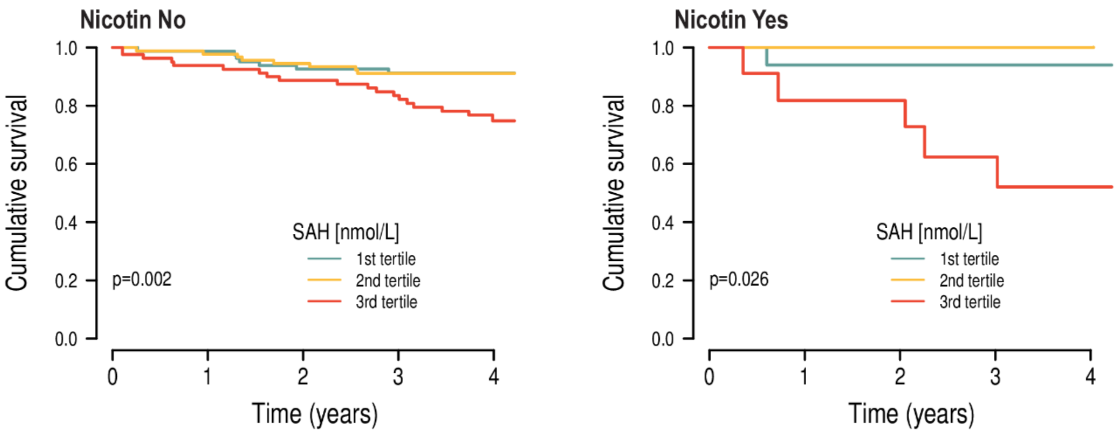

Figure S4: After stratifying the study cohort by their plasma SAH levels into tertiles, higher plasma SAH levels were significantly associated with the primary endpoint in univariate Kaplan-Meier survival analysis in non-smoker ($p = 0.002$) and in smoker ($p = 0.026$).

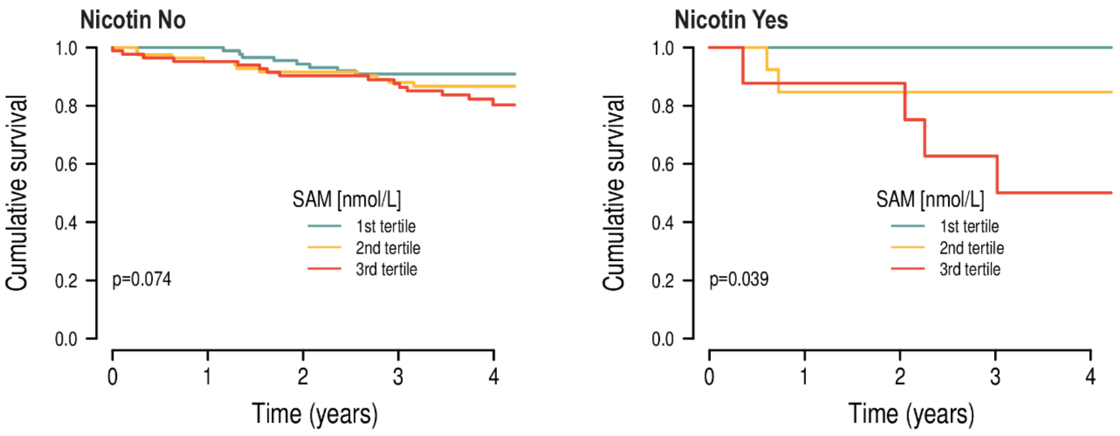

Figure S5: After stratifying the study cohort by their plasma SAM levels into tertiles, higher plasma SAM levels were significantly associated with the primary endpoint in univariate Kaplan-Meier survival analysis in smoker (p = 0.039), but not in non-smoker (p = 0.074).

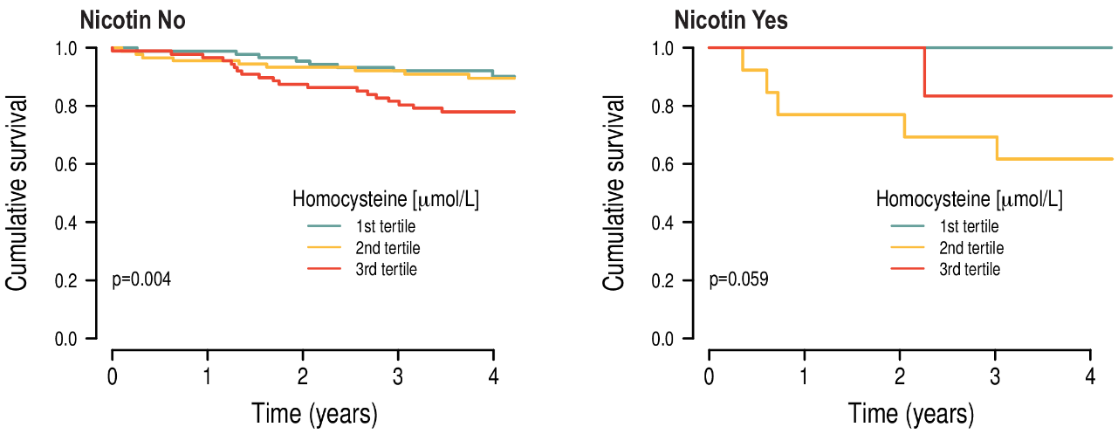

Figure S6: After stratifying the study cohort by their plasma homocysteine levels into tertiles, higher plasma homocysteine levels were significantly associated with the primary endpoint in univariate Kaplan-Meier survival analysis in non-smoker (p = 0.004), but not in smoker (p = 0.059).
